# Development of the Quality of Life Scale for Shift-working Nurses

**DOI:** 10.1016/j.shaw.2024.11.004

**Published:** 2024-11-13

**Authors:** Soner Berşe, Hüseyin Çapuk, Ali Ağar

**Affiliations:** 1 Faculty of Health Sciences, Gaziantep University, Turkey; 2 Vocational School of Health Services, Şırnak University, Turkey; 3 Şavşat Vocational School, Artvin Çoruh University, Turkey

**Keywords:** Nurse, Psychometry, Quality of life, Scale development, Shift work

## Abstract

**Background and aim:**

Shift work is known to have a significant impact on the health and well-being of nurses, and there is a need for practical tools to assess this impact. This study introduces the “Quality of Life Scale for Shift-Working Nurses” (QoLS-SWN), which was developed to provide a reliable and valid tool for assessing the effects of shift work on nurses' health and well-being.

**Methods:**

This study employed a methodological research design to develop and validate a new scale for assessing the quality of life of shift-working nurses. Data were collected from two samples of nurses working in various hospitals. The first sample (*n* = 202) was used for exploratory factor analysis (EFA) to identify the scale's underlying factor structure. The second sample (*n* = 246) was used for confirmatory factor analysis (CFA) to validate the identified structure. The development process included an extensive literature review, expert consultations, and pilot testing. Factor analysis and reliability tests were conducted to ensure that the scale is highly reliable.

**Results:**

The QoLS-SWN demonstrated excellent reliability, with a Cronbach's alpha of 0.95, and factor loadings ranging from 0.56 to 0.90. The scale comprises three dimensions: physical and mental exhaustion, health risks and job safety, and social and psychological interactions, explaining 71.89% of the total variance. CFA affirmed the structural validity of the scale, with fit indices indicating a good model fit (CMIN/df = 2.33, GFI = 0.86, IFI = 0.95, TLI = 0.94, CFI = 0.95, RMSEA = 0.07).

**Conclusion:**

The QoLS-SWN is a reliable and valid tool for measuring the impact of shift work on nurses' quality of life. By providing insights into the multifaceted consequences of shift work, the scale may guide the development of targeted interventions to enhance nurses' quality of life. This scale can inform nursing practice and policy, ultimately improving nurse well-being and patient care outcomes.

## 1 Introduction

In the modern world, nearly one-fifth of the global workforce is engaged in shift work [1]. According to a 2019 study by Eurofound, the proportions of shift workers among all employees are 21% in Europe, 38% in the United States, and 11% in Türkiye, respectively [2]. Shift work is when employees are required to work outside of the standard daytime working hours. This type of work involves a schedule where employees rotate shifts over a designated period, such as a week or a month. Nurses working shifts experience disruptions to sleep-wake cycles, as well as social isolation due to irregular working hours, which may lead to physical and psychological problems [3].

In the healthcare system, shift work is indispensable to ensure uninterrupted patient care. Nurses, who have a key role in delivering healthcare services, work on rotating schedules to provide 24-hour care to patients [1]. Shift work, particularly working night shifts, is a major cause of circadian rhythm disruption, resulting in significant alterations to sleep and biological functions. These changes affect physical and mental health adversely and can exacerbate existing medical conditions [4,5]. In studies involving nurses, night shift work has been associated with sleep disorders, increased family stress, mood changes, and an elevated risk of chronic health problems, such as obesity, diabetes, and cardiovascular disease [6,7]. Shift work disrupts synchronization of the circadian rhythm, posing a higher risk of cardiovascular and metabolic diseases among night shift workers compared to daytime workers. Additionally, working at night can lead to decreased attention and alertness, sleep deprivation, and fatigue, resulting in reduced work efficiency and an increased likelihood of errors and injuries in healthcare interventions [8]. A positive correlation between abnormal eating behaviors and shift work has been shown among nurses [9]. A study on Danish nurses reported that working night shifts increases the risk of diabetes [10]. Furthermore, observations suggest that shift work contributes to an increased prevalence of chronic diseases among nurses [[11], [12], [13]].

As defined by the World Health Organization (WHO), the concept of quality of life (QoL) encompasses various dimensions of well-being, including physical health, psychological state, level of independence, social relationships, personal beliefs, and their relationship to salient features of the environment [1]. Studying QoL is particularly important in the context of shift work, as irregular hours and disruptions to normal circadian rhythm can have a profound impact on these dimensions. Nurses represent a unique population because they often face higher levels of occupational stress, physical and emotional exhaustion, and difficult work schedules than other shift workers. This combination of factors requires a specialized tool to accurately assess QoL specifically designed for nurses. A standardized QoL scale tailored to shift-working nurses can provide critical insights into their challenges and assist in the development of targeted interventions to improve their overall well-being [5,7].

Several scales have been developed to assess the quality of work life and related constructs among healthcare professionals. For example, the Work-Related Quality of Life (WRQoL) scale is a comprehensive tool designed for various professional groups. It evaluates general well-being, work-life balance, and job control, demonstrating high internal consistency, and applicability across different professions [20]. However, the WRQoL lacks detailed assessments specific to shift work, making it less suitable for addressing the unique challenges faced by shift-working nurses. The Professional Quality of Life (ProQOL) scale focuses primarily on the emotional aspects of caregiving professions, evaluating dimensions such as compassion satisfaction, burnout, and secondary traumatic stress. While it demonstrates reliability and internal consistency [21], it does not detail the physical and social impacts of shift work, which are critical for shift-working nurses. The Quality of Nursing Work Life (QNWL) scale includes broad dimensions such as job design, job context, and the work environment, generally evaluating the quality of work life [22]. Despite its applicability, the QNWL lacks a detailed evaluation of the specific health risks associated with shift work. The Copenhagen Psychosocial Questionnaire (COPSOQ) evaluates psychosocial factors in the workplace, including job stress, work-life balance, and social support, with strong internal consistency [23]. However, it does not explicitly address the health risks of shift work in detail.

Given the numerous studies revealing the adverse effects of night shifts on health and the large number of shift-working nurses in healthcare services, the availability of standardized measurement tools for assessing healthy lifestyle behaviors is paramount. Such tools can enhance the quality of care provided and reduce medical errors and injuries in healthcare interventions. Recognizing the critical role of nurses in healthcare systems both in our country and globally, the concept of developing a new scale (QoLS-SWN) has emerged from recent studies on the impact of shift work on nurses. This study was conducted with the aim to develop and validate the Quality of Life Scale for Shift-Working Nurses.

## 2 Materials and methods

### 2.1 Study design

This study employed a methodological research design to develop a scale for assessing the QoL of shift-working nurses. The research involved using the proposed scale to collect data specifically related to the QoL of nurses working shifts.

### 2.2 Participants and sampling

This study was conducted with two separate samples of nurses working in public hospitals in a southeastern province of Türkiye. Sample 1 (*n* = 202) was used for exploratory factor analysis (EFA), while Sample 2 (*n* = 246) was used for confirmatory factor analysis (CFA). Only nurses who had worked in shifts for at least the past year were included in the study. For each nurse, shift-working status was verified through hospital records and confirmation from nursing supervisors. To obtain a truly representative sample, we aimed to include the entire population of shift-working nurses at these hospitals. Therefore, random sampling was not used; instead, we sought to include all eligible nurses. Participation in the study was completely voluntary, and no compensation was provided. Ethical approval was obtained for the study, and all participants signed informed consent. It has been recommended that there should be at least 10 participants for each scale item to demonstrate reliability and validity of a scale [14]. As the draft scale consisted of 26 items, a minimum of 130 participants was required for the analyses, while the study was completed with a total of 448 participants.

#### 2.2.1 Scale development process

The QoLS-SWN development process began with an extensive literature review focusing on shift work and QoL. The review aimed to identify existing scales and various dimensions of QoL affected by shift work. Key sources included peer-reviewed journals, books, and reputable online databases. This review provided a comprehensive understanding of the impact of shift work on physical, mental, and social health of nurses.

Following the literature review, a panel of experts was consulted to refine the scale items. The panel comprised nursing professionals, occupational health experts, and psychometricians. The experts reviewed the first draft of the scale and provided feedback on item relevance, clarity, and comprehensibility [15,16]. Their suggestions were incorporated into the scale to ensure that it accurately reflected multiple aspects of QoL among shift-working nurses.

To quantify content validity, the content validity ratio (CVR) of individual items was calculated using Lawshe's method. Items with low CVR values were removed from the scale to enhance its validity. The scale, initially containing 27 items, was reduced by one item after expert evaluations and CVR calculations.

Pilot testing was then conducted with a small group of shift-working nurses (*n* = 30) to assess the clarity and relevance of the scale items. The pilot test aimed to identify any ambiguities or issues in the scale items and to ensure that the instructions and response options were clear to participants. Feedback from the pilot test participants was used to refine the scale items. The final CVR value for the 26-item scale was 0.85, indicating strong content validity.

Following refinement of the scale items, data were collected from a larger sample of shift-working nurses (*n* = 202). The sample of the pilot study were excluded from the main study. This larger sample allowed for robust statistical analysis and validation of the scale. Exploratory factor analysis (EFA) was conducted to identify the underlying factor structure of the scale. During EFA, six additional items (Items #7, 20, 21, 24, 25, and 26) were removed due to cross-loadings, resulting in a final version of the scale with 20 items. Confirmatory factor analysis (CFA) was then performed on a separate sample of 246 shift-working nurses to confirm the validity of this structure. These analyses helped to finalize the scale, ensuring its reliability and validity.

The final version of the QoLS-SWN consists of 20 items distributed across three sub-scales:

- Physical and mental exhaustion
- Health risks and job safety
- Social and psychological interactions

### 2.3 Data collection tools

Data were collected using paper-based, in-person surveys to ensure accurate and comprehensive responses. The data collection process involved the use of a demographic questionnaire and the QoLS-SWN, allowing for a detailed examination of the effects of shift work on nurses' QoL.

#### 2.3.1 Demographic questionnaire

A set of 10 questions was used to identify demographic characteristics of the participants, including sex, age, education level, and work experience. This information is critical for comparing responses to QoLS-SWN across various sub-populations and understanding demographic variations.

#### 2.3.2 Quality of life scale for shift-working nurses (QoLS-SWN)

The QoLS-SWN was designed through a comprehensive review of the literature [1,[6], [7], [8],^17^], is a 20-item scale with a five-option Likert response format (from strongly agree to strongly disagree). The scale is divided into three main sub-scales that measure the various effects of shift work on nurses.

#### 2.3.3 Physical and mental exhaustion

The “physical and mental exhaustion” subscale of the QoLS-SWN addresses the direct effects of shift work on nurses' physical health and mental state, including constant fatigue, insomnia, and burnout. The primary focus of this dimension is on the physical fatigue and mental stress primarily caused by night shifts and long working hours.

#### 2.3.4 Health risks and job safety

The “health risks and job safety” subscale assesses the potential long-term effects of shift work on nurses' overall health and workplace safety. This dimension evaluates health risks such as cancer, chronic diseases, obesity, the likelihood of workplace accidents, and errors, the risk of occupational diseases. It captures the broad spectrum of health and safety issues associated with shift work.

#### 2.3.5 Social and psychological interactions

The “social and psychological interactions” dimension of the QoLS-SWN examines the effects of shift work on nurses' social life and psychological health. It covers aspects such as relationships with family members and friends, participation in social activities, mood changes, and overall life satisfaction. Additionally, it assesses how shift work schedules affect an individual's emotional and social needs.

### 2.4 Data analysis

The collected data were analyzed using SPSS (Statistical Package for the Social Sciences) and AMOS (Analysis of Moment Structures) software. An exploratory factor analysis (EFA) was conducted using principal component analysis (PCA) as the extraction method, with varimax rotation applied to identify the underlying factor structure. The suitability of the data for factor analysis was assessed using the Kaiser–Meyer–Olkin (KMO) measure and Bartlett's test of Sphericity. The internal consistency of the scale was evaluated using Cronbach's Alpha coefficient.

For confirmatory factor analysis (CFA), various model fit indices were calculated to assess the adequacy of the factor structure identified during EFA. These indices included the chi-square test (χ^2^), Root Mean Square Error of Approximation (RMSEA), comparative fit index (CFI), Tucker-Lewis Index (TLI), and goodness of fit index (GFI), among others.

### 2.5 Ethical approval

Approval for the study was obtained from the ethical committee of a state-owned university on October 17, 2023 (No. 2023/80334). The research was conducted in accordance with the principles of the Declaration of Helsinki. Detailed information about the study purpose and procedures involved were provided to the participants, and their rights were safeguarded throughout the study. Additionally, institutional permission was obtained from the state hospital where the nurses worked on October 27, 2023 (No. E-35694300-449-230479556). Both written and verbal consent were obtained from each participant.

## 3 Results

### 3.1 Characteristics of participants

Two distinct samples were used for the analysis of the QoLS-SWN. Sample 1 consisted of 202 nurses, with the majority being male (52%). The mean age was 29.1 years (standard deviation (SD), 5.1 years), and 66.8% of the participants were aged between 26 and 35 years. Most participants had worked at their current workplace for 1 to 5 years (55.4%) and had a total professional experience of 1 to 5 years (45%). Work patterns varied, with 30.2% working in regular shift schedules and 48.5% working irregular shifts, while 57% reported working more than 48 hours per week. The most common work settings were general wards (24.3%), intensive care units (20.3%), and operating theaters (22.8%).

Sample 2 comprised 246 nurses, 51.63% of whom were female. The mean age was 29.1 years (SD, 5.4 years), and 65.45% of the participants were aged between 26 and 35 years. Regarding tenure at their current workplace, 54.47% had worked there for 1 to 5 years, and 47.15% had a total professional experience of 1 to 5 years. Work patterns showed that 31.30% worked in shift schedules and 44.72% worked irregular shifts. Weekly working hours exceeded 48 hours for 52.85% of the participants. The work settings included general wards (27.24%), intensive care units (19.92%), and operating theaters (19.51%) (Table 1).

**Table 1:** Demographic characteristics of nurses

		Sample 1 (EFA)		Sample 2 (CFA)	
		*n*	%	*n*	%
Sex	Female	97	48	127	51,63
	Male	105	52	119	48,37
Age, years (mean ± SD: 29.1 ± 5.1)	18-25 years	46	22.8	62	25,20
	26-35 years	135	66.8	161	65,45
	36 years or above	21	10.4	23	9,35
Working experience in current job	Less than 1 year	32	15.8	49	19,92
	1-5 years	112	55.4	134	54,47
	6-10 years	39	19.4	44	17,89
	11-15 years	19	9.4	19	7,72
Total working experience in profession	Less than 1 year	16	7.9	23	9,35
	1-5 years	91	45	116	47,15
	6-10 years	68	33.7	79	32,11
	11-15 years	27	13.4	28	11,38
Working pattern	08.00-16.00 (Normal)	43	21.3	59	23,98
	Shift work	61	30.2	77	31,30
	Irregular	98	48.5	110	44,72
Weekly working hours	Less than 40 hours	16	7.9	20	8,13
	40-48 hours	71	35.1	96	39,02
	More than 48 hours	115	57	130	52,85
Working unit	Ward	49	24.3	67	27,24
	Intensive care	41	20.3	49	19,92
	Operating room	46	22.8	48	19,51
	Emergency department	19	9.4	24	9,76
	Outpatient clinic	10	5	15	6,10
	Other	37	18.2	43	17,48

### 3.2 Reliability and exploratory factor analysis results

Analysis of the data collected for the development of the QoLS-SWN revealed a KMO value of 0.93 and a significant Bartlett's test result (*p* < 0.05), indicating an excellent sample adequacy and high internal consistency of the test. The KMO measure assesses the suitability of your data for factor analysis, with values closer to 1.0 indicating a higher level of adequacy. The scale was found to consist of 20 items across three dimensions, explaining 71.89% of the total variance. Factor loadings for the scale items ranged from 0.56 to 0.90, confirming high reliability (Cronbach's Alpha: 0.95, Factor 1: 0.96, Factor 2: 0.91, Factor 3: 0.77) (Table 2). Item-total correlation coefficients ranged from 0.39 to 0.81 (Table 3), indicating strong internal consistency within the scale, as all coefficients were above the 0.30 threshold [18].

**Table 2:** Factor analysis of the quality of life scale for shift-working nurses (QoLS-SWN)

Scale items	Physical and mental exhaustion	Health risks and job safety	Social and psychological interactions
1- I always feel tired.		0.84	
2-I always feel sleep-deprived.		0.84	
3- I constantly feel exhausted.		0.83	
4- I feel that my work performance is declining.		0.73	
5- My physical activities have decreased.		0.68	
6- My social activities have decreased.		0.67	
8- Shift work increases the risk of developing chronic diseases.	0.68		
9- Shift work increases the risk of obesity.	0.68		
10-Shift work negatively affects my appetite and digestive function.	0.64		
11-Shift work negatively affects my eating habits.	0.78		
12- Shift work may increase the risk of workplace accidents.	0.80		
13-Shift work increases the risk of occupational diseases.	0.83		
14-Shift work increases the likelihood of making mistakes.	0.84		
15-Shift work causes more job-related stress.	0.82		
16-Shift work leads to decreased attention levels.	0.81		
17- Shift work reduces the quality of my work.	0.82		
18- Shift work leads to burnout.	0.77		
19- I constantly have arguments with my family and friends because of my shift work.	0.77		
22- I use sleep aids such as over-the-counter or prescription sleeping pills.			0.88
23- I consider quitting my job because of shift work.			0.76

**Explained variance**	39.25	23.24	9.40
**Total variance**	% 71.89		

**Kaiser-Meyer-Olkin measure**	0.932		

**Bartlett's test**	X^2^	3879.369	
	df	190	
	Sig	<0.001	
**Cronbach's Alpha** Total: 0.95	0.96	0.91	0.77

**Table 3:** Item-total score correlation coefficients for the quality of life scale for shift-working nurses

Scale items	*r*
1- I always feel tired.	0.67
2-I always feel sleep-deprived	0.65
3- I constantly feel exhausted.	0.71
4- I feel that my work performance is declining.	0.68
5- My physical activities have decreased.	0.64
6- My social activities have decreased.	0.59
8- Shift work increases the risk of developing chronic diseases.	0.68
9- Shift work increases the risk of obesity.	0.75
10-Shift work negatively affects my appetite and digestive function.	0.57
11-Shift work negatively affects my eating habits.	0.72
12- Shift work may increase the risk of workplace accidents.	0.70
13-Shift work increases the risk of occupational diseases.	0.79
14-Shift work increases the likelihood of making mistakes.	0.81
15-Shift work causes more job-related stress.	0.80
16-Shift work leads to decreased attention levels.	0.82
17- Shift work reduces the quality of my work.	0.80
18- Shift work leads to burnout.	0.80
19- I constantly have arguments with my family and friends because of my shift work.	0.81
22- I use sleep aids such as over-the-counter or prescription sleeping pills.	0.39
23- I consider quitting my job because of shift work.	0.61

**Fig. 1:**
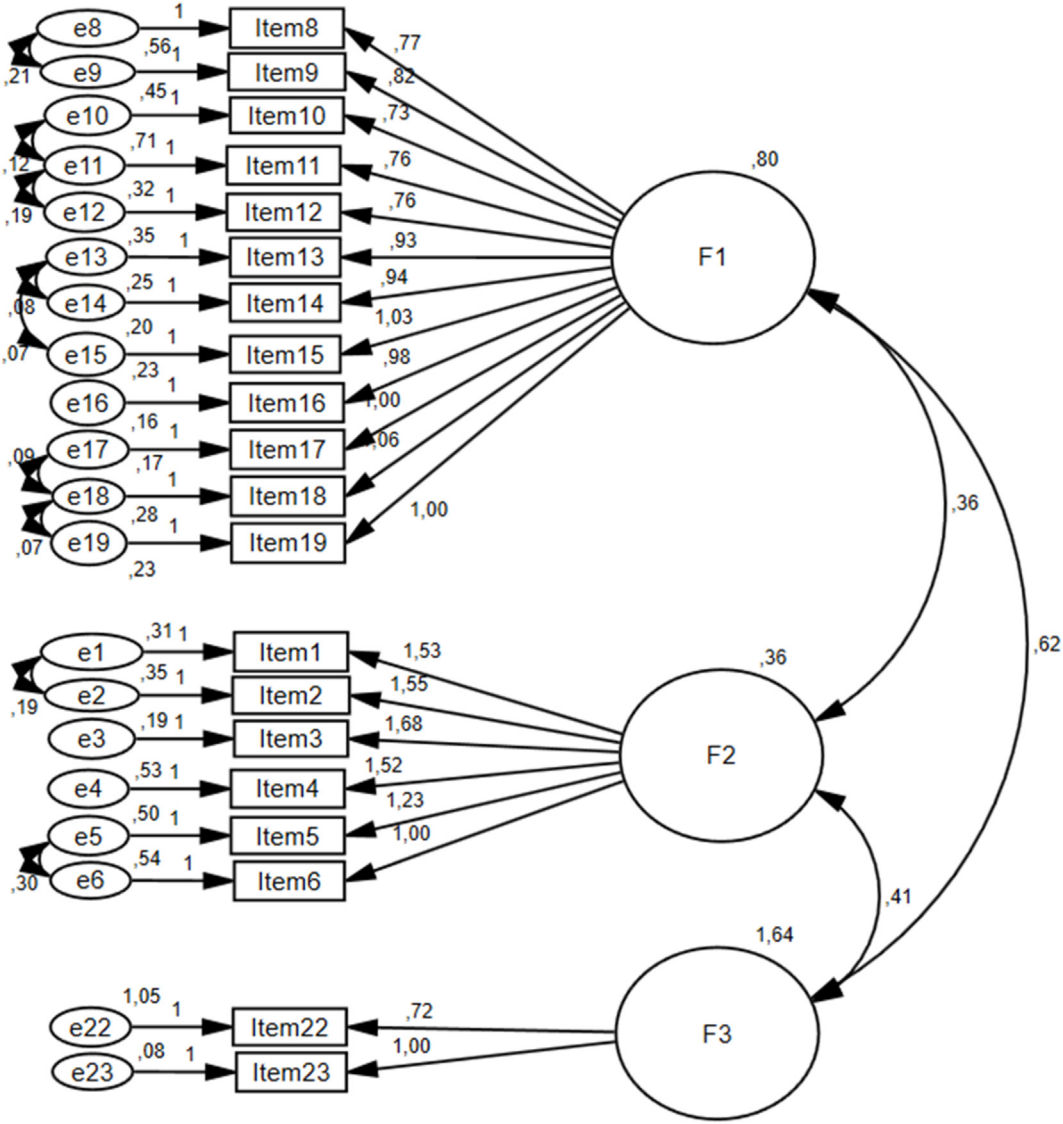
Confirmatory factor analysis model of the QoLS-SWN scale.

A CFA was conducted to evaluate the fit of the QoLS-SWN factors. The analysis identified cross-loadings for six items (#7, 20, 21, 24, 25, and 26), which were subsequently removed to refine the model. Covariance values were reviewed, and variables within the same factor exhibiting the highest values were correlated. Modifications were made by reviewing the modification index scores and correlating variables with the highest modification index (M.I) values, leading to nearly acceptable levels of significance and fit indices for the model (Fig. 1). In the CFA, items' factor loadings and the significance of standardized regression coefficients were assessed to determine the model's adequacy [19]. The standardized regression coefficients for the items were statistically significant (Table 4), confirming that the model with three factors and 20 items, as shown in Fig. 1, fits the data (Table 5). The model's fit was further validated using structural equation modeling following EFA.

**Table 4:** Item/factor load values

Relationships			Standard regression weight	Regression weight	S.E.	C.R.	*p*
Item 16	<---	F1	0.90	0.97	0.04	21.67	˂0.01
Item 15	<---	F1	0.88	1.02	0.05	20.53	˂0.01
Item 14	<---	F1	0.88	0.94	0.04	20.16	˂0.01
Item 13	<---	F1	0.85	0.93	0.04	18.91	˂0.01
Item 12	<---	F1	0.75	0.76	0.05	15.05	˂0.01
Item 11	<---	F1	0.76	0.76	0.04	15.48	˂0.01
Item 10	<---	F1	0.61	0.72	0.06	10.95	˂0.01
Item 9	<---	F1	0.73	0.81	0.05	14.44	˂0.01
Item 8	<---	F1	0.67	0.76	0.06	12.57	˂0.01
Item 17	<---	F1	0.90	1	0.04	21.51	˂0.01
Item 18	<---	F1	0.87	1.05	0.04	23.18	˂0.01
Item 19	<---	F1	0.88	1			
Item 6	<---	F2	0.63	1			
Item 5	<---	F2	0.72	1.23	0.08	14.48	˂0.01
Item 4	<---	F2	0.77	1.51	0.14	10.15	˂0.01
Item 3	<---	F2	0.91	1.68	0.14	11.31	˂0.01
Item 2	<---	F2	0.84	1.54	0.14	10.70	˂0.01
Item 1	<---	F2	0.85	1.53	0.14	10.83	˂0,01
Item 23	<---	F3	0.97	1			
Item 22	<---	F3	0.66	0.71	0.09	7.93	˂0.01

**Table 5:** Fit indices of the model

Fit index	Value after modification	Acceptable fit	Good fit
CMIN/df	2.33	≤5	≤3
GFI	0.86	≥0.85	≥0.90
IFI	0.95	≥0.90	≥0.95
TLI	0.94	≥0.90	≥0.95
CFI	0.95	≥0.95	≥0.97
RMSEA	0.07	≤0.08	≤0.05
NFI	0.92	≥0.90	≥0.95

CFI, comparative fit index; GFI, goodness of fit index; RMSEA, Root Mean Square Error of Approximation; TLI, Tucker-Lewis Index.

## 4 Discussion

This study successfully developed and validated the Quality of Life Scale for Shift-Working Nurses (QoLS-SWN), offering a specialized tool to assess the unique challenges faced by nurses working shifts. By addressing a significant gap in the literature, the scale provides a reliable and valid instrument that holds considerable value for both research and practical applications. The QoLS-SWN demonstrated strong reliability and validity, with high internal consistency and a robust factor structure, indicating that the scale is a reliable tool for assessing the intended constructs.

The QoLS-SWN is divided into three sub-scales: physical and mental exhaustion, health risks and job safety, and social and psychological interactions. These dimensions align with existing literature on the impact of shift work on nurses. Studies have consistently shown that shift work, particularly night shifts, can lead to physical fatigue, burnout (encompassing emotional exhaustion, depersonalization, and reduced personal accomplishment), increased health risks, and disruptions to social and psychological well-being. For example, Ferri et al (2016) highlighted the psychological and physical health issues faced by nurses working rotating night shifts, reinforcing the relevance of these dimensions. It should be noted that the QoLS-SWN assesses nurses' perceptions of the health risks associated with shift work rather than measuring the actual health risks. This distinction is crucial because the perception and concern about health risks can adversely affect nurses' well-being.

Our scale, the QoLS-SWN, consists of three dimensions, including physical and mental exhaustion, health risks and job safety, and social and psychological interactions. These sub-scales provide a comprehensive evaluation of shift work's impact on nurses. The scale's high Cronbach's alpha value (0.95) and the high internal consistency values of its sub-scales (0.96, 0.91, and 0.77) demonstrate its reliability. Furthermore, the QoLS-SWN explains 71.89% of the total variance, indicating that its factor structure is robust and well represents the underlying data.

As outlined in the Introduction, existing tools often fail to capture the unique challenges experienced by this population, which can lead to inadequate assessment and intervention strategies. To gain a deeper understanding of the contributions and potential applications of the QoLS-SWN, we compared it with other commonly used scales in the field. Psychometrically, there are some notable similarities and differences between the QoLS-SWN and those scales. When comparing the QoLS-SWN to those other scales, our scale offers a more targeted and appropriate tool for assessing the work-related QoL of shift-working nurses. The ProQOL focuses on emotions and does not address the physical and social impacts of shift work, and therefore, the QoLS-SWN is better suited to identify the specific needs of shift-working nurses. The QNWL does not assess specific health risks associated with shift work. By comparison, the QoLS-SWN offers a unique contribution in addressing these specific risks. The COPSOQ does not specifically address potential health hazards associated with shift work. In contrast, the QoLS-SWN provides a valid and reliable tool for this purpose by focusing on specific health risks and job safety related to shift work.

Introducing the QoLS-SWN, this study fills an important gap by providing a comprehensive instrument that can accurately reflect the multifaceted impact of shift work on nurses' lives.

The QoLS-SWN has significant implications for nursing practice and healthcare policy. It offers a reliable measure of quality of life for shift-working nurses, helping healthcare organizations identify areas for targeted interventions. These interventions can address issues such as physical and mental exhaustion, mitigate health risks, and enhance social and psychological well-being. By implementing flexible scheduling, providing mental health resources, and fostering a supportive work environment, healthcare organizations can improve nurses' well-being and job satisfaction.

In summary, the QoLS-SWN's robust psychometric properties and well-defined structure address the specific needs of shift-working nurses, distinguishing it from other scales commonly used in the field. Its comprehensive evaluation of shift work conditions makes it a powerful tool for assessing their effects. These features provide a strong foundation for the QoLS-SWN's broad application in both research and practical settings.

## 5 Conclusion

The Quality of Life Scale for Shift-Working Nurses (QoLS-SWN), developed and validated in this study, can effectively address the unique challenges faced by nurses working shifts. The scale has strong psychometric properties with high reliability and validity.

The QoLS-SWN can guide the development of workplace policies that promote a healthier work environment, potentially leading to better job satisfaction, reduced turnover, and improved patient care outcomes. Future research efforts should focus on further refining and validating the scale in diverse contexts to ensure its effectiveness in addressing the needs of shift-working healthcare professionals. To better understand the long-term impact of shift work on quality of life among nurses, a longitudinal study design could be considered in future research.

### 5.1 Limitations

This study has several limitations that should be acknowledged and considered for future research. Firstly, the sample was limited to nurses working in hospitals in the southeastern region of Turkey, which may limit the generalizability of the findings to other regions or countries.

Secondly, the cultural context of the study is a significant limitation. Cultural factors can influence how nurses perceive and report their quality of life. Therefore, to ensure the validity of the QoLS-SWN in different cultural settings, it is important to perform translation and cross-cultural psychometric testing of the scale.

Thirdly, the QoLS-SWN consists of 20 items, which may lead to response fatigue among participants, potentially impacting the quality of responses. A shorter version of the scale might be more practical for large-scale use, especially in time-constrained environments such as hospitals. Therefore, future studies should consider developing a shorter version of the scale without compromising its psychometric properties.

Additionally, the use of paper-based in-person surveys may introduce interviewer bias or social desirability bias. Future research may consider using more anonymous data collection methods to reduce such biases. Finally, we did not assess convergent and divergent validity by comparing the QoLS-SWN with other established scales. This is a limitation of our study, and future research should include such analyses to further establish the validity of the scale.

## CRediT authorship contribution statement

**Soner Berşe:** Writing – review & editing, Writing – original draft, Methodology, Conceptualization. **Hüseyin Çapuk:** Writing – original draft, Methodology, Conceptualization. **Ali Ağar:** Writing – original draft, Visualization, Validation, Methodology, Conceptualization.

## Disclosure of grants or other funding

This research received no specific grant from any funding agency in the public, commercial, or not-for-profit sectors.

## Conflicts of interest

There are no conflicts of interest to declare.
